# Toxicity Assessment of Catechins on Representative Aquatic Organisms and Terrestrial Plant

**DOI:** 10.3390/toxins17050244

**Published:** 2025-05-14

**Authors:** Khawaja Muhammad Imran Bashir, Hye-Ryeon An, Bertoka Fajar Surya Perwira Negara, Gabriel Tirtawijaya, Maria Dyah Nur Meinita, Jae-Hak Sohn, Dicky Harwanto, Jae-Suk Choi

**Affiliations:** 1 Department of Seafood Science and Technology, The Institute of Marine Industry, Gyeongsang National University, Tongyeong 53064, Republic of Korea; imranbashir@gnu.ac.kr (K.M.I.B.); grace@gnu.ac.kr (H.-R.A.); 2 German Engineering Research and Development Center, LSTME-Busan Branch, Busan 46742, Republic of Korea; 3 Department of Marine Science, University of Bengkulu, Jl. W.R Soepratman, Bengkulu 38371, Indonesia; ftrnd12@silla.ac.kr; 4 Faculty of Biotechnology, University of Surabaya, Jalan Raya Kalirungkut Surabaya, Surabaya 60292, Indonesia; gabrieltirtawijaya@staff.ubaya.ac.id; 5 Faculty of Fisheries and Marine Science, Jenderal Soedirman University, Purwokerto 53123, Indonesia; maria.meinita@unsoed.ac.id; 6 Center for Maritime Bioscience Studies, Jenderal Soedirman University, Purwokerto 53123, Indonesia; 7 Department of Food Science and Culinary Arts, College of Health and Welfare, Silla University, Busan 46958, Republic of Korea; jhsohn@silla.ac.kr; 8 Faculty of Fisheries and Marine Science, Diponegoro University, Semarang 50275, Indonesia

**Keywords:** algae, cell density, eco-friendly, germination, plankton, toxicity

## Abstract

Catechins, renowned for their health benefits, have unexamined environmental impacts. This study assessed the toxicity of crude catechin and catechin hydrate on invertebrate larvae, plant, and microalgae. The survival rates of *Daphnia magna* Straus and *Artemia salina* L. were monitored every 24 h over a three-day period. The germination rate and radicle length of *Lactuca sativa* L. was measured every 24 h for four days. Inhibitory effects were evaluated in both freshwater and seawater cultures of *Chlorella vulgaris* Beijerinck, with cell density recorded every 24 h and yield inhibition calculated after 96 h. Results indicated that increasing catechin concentration and exposure duration decreased the survival rate of *D. magna* and *A. salina*. *Daphnia magna* was more sensitive to catechins than *A. salina*, with 24 h lethal concentration 50 (LC-50) values of 1174 µg/mL compared to 1895 µg/mL for crude catechin, and 54 µg/mL compared to 153 µg/mL for catechin hydrate. The germination rate and radicle length of *L. sativa*, along with the cell density of *C. vulgaris*, decreased with increasing catechin concentration, but remained higher even after prolonged exposure. At low catechin concentrations, *C. vulgaris* cell density exceeded control levels. This study demonstrates that catechins in aquatic environments can significantly impact ecosystems. At certain concentrations, catechins are toxic and potentially lethal to aquatic organisms. Conversely, at lower concentrations, catechins may promote microalgal growth, suggesting a fertilizing effect. Understanding these dynamics is crucial for maintaining the stability of aquatic ecosystems.

## 1. Introduction

Catechins are a type of flavonoid, which are plant polyphenols that include epicatechin (EC), epigallocatechin (EGC), epicatechin gallate (ECG), and epigallocatechin gallate (EGCG) as diastereoisomers [1]. Among these, catechin hydrate is a natural flavonoid known for its antioxidant and anti-inflammatory properties. It is beneficial not only for prevention but also for the treatment of diseases caused by oxidative damage, such as inhibiting cancer growth [2,3]. As important dietary components, catechins are found in plants like green tea, nuts, chocolate, red wine, and fruits such as apricots, black grapes, cocoa, and cherries [4,5]. These compounds serve as antioxidants, antimicrobials, and anticarcinogens [6,7]. Their efficiency as antioxidants surpasses many other substances, making them a cost-effective choice in food production [8]. Studies on both animals and humans have demonstrated that catechins impact lipid metabolism by reducing triglycerides, total cholesterol [9], and body fat accumulation [10,11,12]. By binding strongly to reactive oxygen species (ROS), they absorb free radicals [1]. As environmentally friendly plant polyphenols [13], even minimal doses can lead to health improvements [14].

The toxic effects of catechins on aquatic environments need to be evaluated. Freshwater and saltwater ecosystems have distinct characteristics that influence the sensitivity of their resident biota. Studies on the effects of catechins on both freshwater and marine organisms are crucial in understanding the broader ecological risks. In the present study, we utilized several organisms: *Daphnia magna* Straus neonates, *Artemia salina* L. nauplii, *Lactuca sativa* L. seeds, and *Chlorella vulgaris* Beijerinck. *D. magna* is a commonly used freshwater organism in environmental studies [15,16], particularly for assessing drug toxicity [17,18,19,20]. It is chosen for toxicity measurement due to its sensitive behavioral and physiological responses to environmental factors and chemicals, making it a biomarker in studies on phenotype, toxicology, and life cycle evolution [21]. *Daphnia magna* is a popular freshwater organism for bioassays in biological and environmental studies, with *A. salina* serving as the seawater equivalent. This species is used in food safety [22], medical safety [23], therapeutic and pharmacological [24,25,26], and eco-toxicity studies [15,16,27,28].

*Lactuca sativa* is a macro plant frequently used in bioassays and environmental quality bioindicator studies due to its quick and easy germination [29]. Its sensitivity to environmental changes makes it suitable for water [30] and soil quality studies [22], as well as studies on heavy metal extraction [31] and air pollution [32]. *Lactuca sativa* has also been used in bioherbicide [33] and allelochemical studies [34]. Microalgae, another aquatic organism, is useful for monitoring environmental pollution and conducting aquatic toxicity tests [16,35]. *Chlorella vulgaris* is economically and effectively used in toxicity research due to its sensitivity to toxicants and ease of laboratory cultivation [16].

Previous studies have indicated that catechins are more toxic to Gram-positive bacteria (*Bacillus subtilis*) than to Gram-negative bacteria (*Escherichia coli*) [1]. Additionally, high doses of catechins can cause liver side effects, though this is not the case when consumed as brewed tea, as beverage extracts, or in food form [36]. The appropriate concentration of catechins has been shown to reduce mortality in brine shrimp when exposed to toxic methanol concentrations [37].

*Daphnia magna* and *A. salina* are crucial components of aquatic ecosystems. *Daphnia magna*, found in freshwater, feeds on algae and phytoplankton, helping to control algal blooms (eutrophication) and serving as a vital food source for fish and invertebrates. *Artemia salina*, which inhabits brackish water and seawater, consumes microorganisms and detritus, contributing to ecosystem balance. It also supports the carbon and nitrogen cycles and is an important food source for fish and invertebrates. Both species play key roles in the aquatic food web and help maintain ecological stability.

*Lactuca sativa*, although not an aquatic macrophyte, shows potential for use in aquatic systems through its phytoremediation abilities. In terrestrial–aquatic integrated systems, it can absorb inorganic nitrogen compounds (e.g., nitrates) and other pollutants from aquatic organism waste, helping stabilize water chemistry and improve system efficiency. *Chlorella vulgaris*, a single-celled microalga, is a primary producer in aquatic environments. Through photosynthesis, it generates oxygen and organic matter that support the food chain. It also absorbs excess nutrients like nitrogen and phosphate, reducing eutrophication, and can capture heavy metals and toxins, making it effective for bioremediation in polluted waters.

By conducting this study, we aim to generate basic knowledge about the significant impacts of catechins on the stability of aquatic ecosystems. Understanding these impacts is essential, as catechins, whether naturally occurring or arising through environmental exposure, can potentially disrupt ecological balance, affect species survival, and alter water quality. This study is expected to contribute to environmental risk assessment and inform future strategies for managing the presence of these compounds in aquatic environments.

## 2. Results

### 2.1. HPLC Analysis of Crude Catechin and Catechin Hydrate

**Figure 1:**
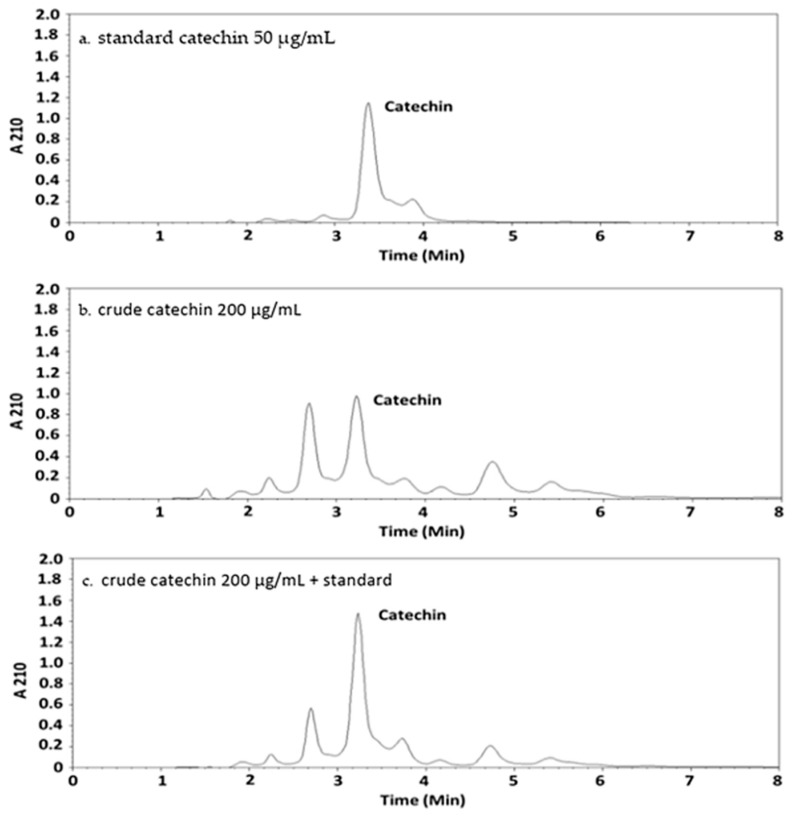
HPLC chromatogram of standard catechin at 50 µg/mL (**a**), crude catechin at 200 µg/mL (**b**), and combination of crude catechin (200 µg/mL) + standard catechin (50 µg/mL) (**c**).

For the high-performance liquid chromatography (HPLC) analysis, catechin hydrate standard (50 µg/mL), crude catechin (200 µg/mL), and a combination of both were injected into the HPLC instrument. The resulting chromatogram is shown in Figure 1. Catechin retention times were observed to range from 3.0 to 3.5 min.

Catechin was successfully identified in the extract samples. In the chromatogram of the pure catechin hydrate standard (Figure 1a), a distinct peak was observed at a retention time characteristic of catechin. The extract sample (Figure 1b) exhibited a peak at the same retention time, indicating the presence of catechin. To further confirm this identification, a co-injection of the extract and catechin standard was performed (Figure 1c). The resulting chromatogram displayed a single intensified peak at the same retention time, with no peak splitting or additional signals. This co-elution demonstrated that the compound in the extract matched the standard completely. This confirms the identity of the compound as catechin, showing qualitative agreement and chromatographic consistency, and providing strong evidence for its presence in the extract.

### 2.2. Larvicidal Effects of Crude Catechin and Catechin Hydrate on the Survival of D. magna

The larvicidal effects on *D. magna* neonates are detailed in Table 1. The control group of neonates exhibited high survival rates of 100%, 94%, and 85% at 24, 48, and 72 h, respectively.

Crude catechin showed a larvicidal effect at concentrations of 2.0 and 3.0 mg/mL after 24 h, with the most significant effect observed at 3.0 mg/mL, resulting in only 10% survival. The lethal concentration 50 (LC-50), which is the concentration required to kill 50% of the organisms, was determined to be 1174 µg/mL at 24 h. At a concentration of 0.5 mg/mL, crude catechins showed a mild larvicidal effect, with survival rates of 43% at 48 h and 28% at 72 h. At 48 and 72 h, no neonates survived at concentrations of 2.0 or 3.0 mg/mL.

Catechin hydrate demonstrated the most severe larvicidal effect at a concentration of 0.5 mg/mL after 24 h, with 0% survival. At a concentration of 0.1 mg/mL for 24 h, the survival rate was 38%. The LC-50 at 24 h was found to be 54 µg/mL. After 72 h, all tested concentrations showed a larvicidal effect. Specifically, a concentration of 0.01 mg/mL resulted in a 40% survival rate, while 0.05 mg/mL resulted in a 20% survival rate. The LC-50 at 72 h was determined to be 7 µg/mL.

### 2.3. Larvicidal Effects of Crude Catechin and Catechin Hydrate on the Survival of A. salina

In this study, pure seawater was used as a negative control and did not kill *A. salina* after 24 h, with minimal mortality observed at 48 h (2%) and 72 h (7%). The larvicidal effects of crude catechin on *A. salina* nauplii are detailed in Table 2. After 24 h, a concentration of 0.5 mg/mL resulted in 100% survival, but higher concentrations induced mortality, with 3.0 mg/mL causing the highest mortality rate of 84%. The LC-50 at 24 h was determined to be 1895 µg/mL. At 48 h, concentrations of 2.0 and 3.0 mg/mL resulted in no surviving *A. salina* nauplii, while a concentration of 1.0 mg/mL had a low survival rate of 20%.

Catechin hydrate showed larvicidal effects at concentrations of 0.1 and 0.5 mg/mL after 24 h, with survival rates of 45% and 40%, respectively. The LC-50 at 24 h was 153 µg/mL. After 72 h, *A. salina* nauplii exposed to concentrations of 0.01 and 0.05 mg/mL showed survival rates of 42% and 23%, respectively. The LC-50 at 72 h was determined to be 9 µg/mL.

### 2.4. Inhibitory Effect of Crude Catechin and Catechin Hydrate on Germination of L. sativa

The toxicity and inhibition levels of crude catechin and catechin hydrate on the germination and radical growth of *L. sativa* are summarized in Table 3. *Lactuca sativa* seeds treated with 1.0 mg/mL crude catechin exhibited a higher germination rate of 87.5% compared to the control at 24 h. However, at a concentration of 50.0 mg/mL, germination did not occur. At 1.0 mg/mL, radicle growth was slower than that of the control, with lengths of 3.11 mm compared to 3.84 mm for the control. At 10.0 mg/mL, germination was 35% at 24 h and 75% at 96 h, both lower than that of the control.

Catechin hydrate resulted in longer radicle growth at concentrations of 0.1, 0.5, and 1.0 mg/mL. At 0.1 mg/mL, radical length was 4.47 mm at 24 h, compared to 4.10 mm for the control. At 5.0 mg/mL, radical length was 2.95 mm. After 48 h, a concentration of 0.1 mg/mL catechin hydrate resulted in a radical length of 13.26 mm, compared to 15.74 mm for the control. Germination rates were lower for catechin hydrate, with an 83.3% germination rate at 0.1 mg/mL compared to 93.3% for the control at 96 h.

### 2.5. Toxicity of Crude Catechin and Catechin Hydrate on Cell Density of Freshwater C. vulgaris

The effects of crude catechin and catechin hydrate on the cell density of freshwater *C. vulgaris* are detailed in Table 4. After 24 h, crude catechin at concentrations of 0.5 and 1.0 mg/mL significantly increased cell density, with values of 1.50 × 10^6^ cells/mL and 1.42 × 10^6^ cells/mL, respectively, compared to the control at 1.16 × 10^6^ cells/mL. However, a concentration of 2 mg/mL showed no significant growth enhancement. At 72 h, a concentration of 1.0 mg/mL resulted in a lower cell density than that of the control, while 0.5 mg/mL showed no significant difference. Yield inhibitions for catechin hydrate were 12.29 ± 1.60% and 33.84 ± 0.60% at concentrations of 1.0 and 2.0 mg/mL, respectively, with lower concentrations showing approximately a four-fold increase in yield.

Catechin hydrate increased cell density at all concentrations after 24 h, with values ranging from 1.33 × 10^6^ to 1.59 × 10^6^ cells/mL. From 48 h to 96 h, a concentration of 1.0 mg/mL did not exceed the cell density of the control. From 72 h to 96 h, a concentration of 0.5 mg/mL did not exceed the control cell density, but lower concentrations resulted in increased cell density.

### 2.6. Toxicity of Crude Catechin and Catechin Hydrate on Cell Density of Seawater C. vulgaris

The effects of crude catechin on the cell density of seawater *C. vulgaris* are presented in Table 5. After 24 h, there were no significant changes in cell density compared to the control. However, significant changes were observed at 48 h, with concentrations of 0.5 and 2.0 mg/mL resulting in cell densities of 1.90 × 10^6^ and 1.37 × 10^6^ cells/mL, respectively. By 96 h, all seawater *C. vulgaris* treatments showed significantly different cell density values. Concentrations of 1.0 and 2.0 mg/mL resulted in lower cell density values (2.63 × 10^6^ and 1.61 × 10^6^ cells/mL, respectively) compared to that of the control (3.96 × 10^6^ cells/mL). At 0.5 mg/mL, cell density increased by 2.61%, while concentrations of 1.0 and 2.0 mg/mL led to inhibitions of 19.90% and 35.26%, respectively.

When exposed to catechin hydrate, seawater *C. vulgaris* showed significant changes in cell density compared to the control after 24 h at all concentrations. The lowest concentration resulted in a cell density of 1.36 × 10^6^ mg/mL, while at 0.1 mg/mL, cell density was 1.56 × 10^6^ mg/mL. Higher concentrations (0.5 and 1.0 mg/mL) decreased cell density to 1.35 × 10^6^ and 1.32 × 10^6^ cells/mL, respectively. After 48 h, concentrations of 0.05 and 0.1 mg/mL showed no significant differences in cell density (2.21 × 10^6^ and 2.20 × 10^6^ cells/mL) compared to the control (2.29 × 10^6^ mg/mL). After 96 h, all concentrations except 0.1 mg/mL were significantly different from that of the control. Yield inhibitions for concentrations of 0.5 and 1.0 mg/mL were 16.37 ± 1.53% and 35.97 ± 1.70%, respectively, while lower concentrations (0.05 and 0.1 mg/mL) increased cell density by 5.92% and 1.71%, respectively.

## 3. Discussion

In this study, *D. magna* was placed on 24-well plates with 2 mL of solution, minimizing the use of compounds and bioactive reagents. Crude catechins showed mild larvicidal effects at 2.0 mg/mL and high larvicidal effects at 3.0 mg/mL after 24 h, with survival rates of 45.0 ± 4.1% and 10.0 ± 4.1%, respectively (LC-50 at 24 h: 1174 µg/mL). At 48 h, 0.5 mg/mL showed mild effects, while 1.0 mg/mL showed high effects, and 2.0 and 3.0 mg/mL resulted in 100% mortality. Catechin hydrate at 0.5 mg/mL resulted in 0% survival at 24 h (LC-50 at 24 h: 54 µg/mL). At 0.1 mg/mL, it had mild larvicidal effects, with a survival rate of 38.0 ± 2.9%. The increase in mortality is likely due to the higher concentrations and longer exposure times [38]. Catechin hydrate was more toxic to *D. magna* than crude catechin, consistent with Hu et al. [36], who found fewer side effects from catechins in brewed tea or food than in pure form.

For *A. salina* nauplii, crude catechins at 0.5, 1.0, and 2.0 mg/mL did not show larvicidal effects up to 24 h, while 3.0 mg/mL did show effects (LC-50 at 24 h: 1895 µg/mL). At 48 h, 2.0 and 3.0 mg/mL resulted in 100% mortality, with 1.0 mg/mL showing mild effects (20.0 ± 4.1% survival rate). Catechin hydrate was more toxic to *A. salina* than crude catechin, with mild effects at 0.1 and 0.5 mg/mL after 24 h (survival rates of 45.0 ± 5.0% and 40.0 ± 5.0%, respectively). The LC-50 at 24 h was observed to be 153 µg/mL. At 48 h, 0.01 and 0.05 mg/mL showed no larvicidal effects, with a survival rate of 80% and 52%, respectively. The LC-50 at 48 h was 70 µg/mL. After 72 h, 0.01 mg/mL showed mild larvicidal effects, and 0.05 mg/mL showed high effects, with survival rates of 42% and 23%, respectively. The LC-50 at 72 h was noted to be 9 µg/mL.

Increasing both the catechin concentration and the exposure time showed reduced survival rates of *D. magna* and *A. salina*. Catechin hydrate was more toxic, likely due to its pure form and greater concentration of active compounds, resulting in stronger biological effects. Its higher solubility and bioavailability further enhance absorption by organisms, which intensifies its toxic impact. In contrast, crude catechin, being a crude extract, contains a mixture of various compounds, some of which may function as buffers or neutralizers of toxic effects [22,39]. The absence of these mitigating components in catechin hydrate could contribute to its more pronounced toxicity in zooplankton.

The larvicidal effect of crude catechin on *A. salina* was higher than that reported in Rodrigues et al. [39], who reported no toxic effects of *Camellia sinensis* L. Kuntze (green tea) on *A. salina* up to 1.0 mg/mL for 48 h, but lower than that reported in Silva et al. [22], who reported mild larvicidal effects of matcha green tea extract (*C. sinensis*), with an LC-50 at 24 h of 0.4 mg/mL. In addition, they observed an increase in mortality with increasing concentration (up to 1000 μg/mL) of matcha green tea extract. The larvicidal effects of crude catechin and catechin hydrate on *A. salina* appeared to be weaker than that on *D. magna*. This is probably because *Daphnia* sp. is more sensitive to environmental toxicity than *Artemia* sp., as shown by Ivorra et al. [40] and de la Vega et al. [15]. Harwanto et al. [16] also reported higher mortality in *D. magna* than in *A. salina* when exposed to the same concentrations of crude phlorotannin or pure phlorotannin.

Crude catechin also impacted *L. sativa* germination and radicle growth. Germination was relatively higher at 1.0 mg/mL (87.5 ± 15.0%) but decreased with higher concentrations, with significant inhibition observed at 50.0 mg/mL, resulting in 0% germination. Radicle growth was optimal at 1.0 mg/mL (18.02 ± 3.42 mm) and minimal at 10.0 mg/mL (4.71 ± 1.03 mm). Catechin hydrate led to faster radicle growth at 0.1 mg/mL (29.75 ± 1.00 mm at 96 h). The germination rate decreased with increasing concentrations, with the highest rate at 0.5 mg/mL (90.0 ± 0.00%) and the lowest at 10.0 mg/mL (33.3 ± 15.3%) at 96 h. This pattern suggests that higher catechin levels exert an inhibitory effect on early plant development. However, despite prolonged exposure, both germination rate and radical length remained elevated, indicating a potential resilience or adaptive physiological response in *L. sativa*, enabling partial recovery or continued growth even under suboptimal conditions. A previous study by Harwanto et al. [16] using phlorotannin and phloroglucinol showed lower survival rates for *L. sativa* compared to the present study, suggesting a lower toxicity of crude catechin and catechin hydrate. In this study, radical growth was slower for crude catechin but faster for catechin hydrate compared to phlorotannin and phloroglucinol.

Crude catechin inhibited the cell density of freshwater *C. vulgaris* at all tested concentrations after 24 h. At 48 h, the lowest concentration of catechin hydrate did not show significant changes. At 72 h, 0.5 mg/mL crude catechin and 0.05 and 0.1 mg/mL catechin hydrate increased cell density by 4.61%, 8.10% and 2.91%, respectively. This study found that *C. vulgaris* cell density decreased with increasing catechin concentration, indicating an inhibitory effect of catechin exposure. However, cell density continued to rise with longer exposure, suggesting a potential tolerance of *C. vulgaris* to catechin exposure. At the lowest catechin concentration, cell density was higher than in the control, indicating a potential stimulatory effect of catechin. Rashidinejad et al. [14] reported comparable results, showing that minimal doses of catechins can improve health, while higher doses can be toxic. Seawater *C. vulgaris* exposed to crude catechin and catechin hydrate showed similar patterns. Concentrations of 0.5 mg/mL crude catechin and 0.05 and 0.1 mg/mL catechin hydrate increased cell density by 2.61%, 5.92%, and 1.71%, respectively. A previous study by Harwanto et al. [16] also reported higher toxicity for phlorotannin and phloroglucinol compared to crude catechins and catechin hydrate. The results of the present study suggest that crude catechins and catechin hydrates can act as fertilizers at lower concentrations.

Catechins can induce oxidative stress by promoting the generation of reactive oxygen species (ROS), leading to cellular damage in various biological systems. This oxidative stress affects critical cellular components such as DNA, proteins, and lipids. In aquatic organisms like *D. magna* and *A. salina*, this oxidative stress may contribute to the observed toxicity, as both species serve as valuable models for assessing the overall cytotoxicity of compounds [15,16,17,18,19,21,22,23,24,27,40]. These crustaceans are commonly used to evaluate the potential toxic effects of chemicals, providing insights into the compound’s impact on multiple biological systems within an organism. The primary target organs and tissues for catechins in *D. magna* and *A. salina* include the digestive system, nervous system, reproductive organs, and epidermal tissues. The effects of catechins on these organs suggest that they may disrupt a wide range of physiological processes, leading to broader systemic toxicity. The observed increase in mortality of *D. magna* and *A. salina* with increasing catechin concentration and exposure time in this study is strongly suspected to be associated with elevated levels of ROS, which are known to induce cellular damage across various biological systems [21,22,23,24,27,40]. This finding is consistent with previous studies demonstrating that ROS levels tend to rise in response to higher concentrations and prolonged exposure to a variety of chemical agents. Similar patterns of ROS-induced toxicity have been reported with exposure to nano-titanium dioxide [15], extract of matcha green tea [22], dental restoration materials [23], and extract of *Hyptis suaveolens* [24].

Catechin compounds exhibited a range of phytotoxic effects on *L. sativa* and *C. vulgaris* primarily through their interaction with ROS and their influence on cellular processes. These compounds can act as both protective agents and phytotoxins, depending on their concentration. As phytotoxins, catechins induce ROS production, leading to oxidative stress and cell death in plants and algae. In *L. sativa*, the root system is a primary target of phytotoxins, where catechins or other phytotoxic compounds induce ROS production, leading to root cell death and inhibition of root growth [31,32,33,34,41]. In *C. vulgaris*, phytotoxins primarily target body cells or newly developing cells, especially those involved in cell division or growth processes [16,35,42]. Phytotoxins like those derived from catechins can induce ROS production, causing oxidative stress, damaging cell membranes, and ultimately disrupting cellular functions. They also affect chloroplasts, the main organelles responsible for photosynthesis, which can lead to cell death.

Research on the effects of catechins on aquatic organisms holds enormous potential to provide insights into how these natural compounds can impact aquatic ecosystems and environmental quality. Catechins are known for their various biological benefits, such as antioxidants, antimicrobial, and anti-inflammatory properties [1,2,3,4,5,6,7,8]. However, their effects on aquatic microorganisms, such as bacteria, algae, and protozoa, can vary significantly depending on the concentration, type of microorganism, and environmental quality. Some studies suggest that catechins have the potential to inhibit the growth of certain microorganisms, functioning as natural antimicrobial agents [22,39]. However, at certain concentrations, catechins may disrupt the microbial balance within the aquatic ecosystem, either by reducing microbial diversity or affecting specific populations crucial for biogeochemical cycles. This research demonstrates that crude catechins and catechin hydrate can influence the presence of zooplankton and algae, which are foundational to the aquatic food chain. Their impact on algae, whether through stimulating or inhibiting growth, affects oxygen production in the water and the stability of the aquatic ecosystem. A reduction in zooplankton populations could disrupt the ecological balance and decrease food availability for other organisms in the aquatic food web. The study also indicates that if catechins are applied in high concentrations or over extended periods [38,39], their effects on aquatic microorganisms may become more pronounced. If microorganisms beneficial to water quality, such as organic matter decomposers, are affected, it could interfere with processes like organic matter decomposition and nitrification, both of which are crucial for maintaining nutrient balance in aquatic environments.

It is important to note that further research is needed to better understand the bioaccumulation of catechins in the tissues of microorganisms, as well as the long-term impacts on aquatic microorganisms and the entire ecosystem. If catechins are used in large quantities, potential risks may include changes in the microbial community structure within aquatic environments, ultimately affecting the health and sustainability of the overall aquatic ecosystem. Additionally, the interactions between catechins and other pollutants or chemicals in the water need further exploration, as these compounds may have synergistic or antagonistic effects that could either exacerbate or improve environmental conditions.

## 4. Conclusions

This study demonstrates that the survival rate of zooplankton in both freshwater and seawater decreases with higher concentrations of and longer exposure times to catechin. In plants such as *L. sativa*, germination and radicle growth can still increase with extended catechin exposure, similar to the response in microalgae like *C. vulgaris*, which can increase its cell density even as catechin exposure time extends. *Chlorella vulgaris* can even grow faster than the control at low catechin concentrations, indicating a stimulatory effect of catechins. The use of *A. salina*, *D. magna*, *L. sativa*, and *C. vulgaris* in toxicity tests confirmed the larvicidal and inhibitory effects of various concentrations of crude catechins and catechin hydrate. Crude catechin and catechin hydrate were more larvicidal for *D. magna* than *A. salina*, while the inhibitory effect was greater in seawater *C. vulgaris* compared to freshwater *C. vulgaris*. The results of this study suggest that catechins, at appropriate concentrations, can function as fertilizers, enhancing the growth of terrestrial plants and microalgae.

## 5. Materials and Methods

### 5.1. Sample Preparation

*Daphnia magna* Straus were obtained from a local live feed breeder for fish. Adult *D. magna* were reared in a 2 L Erlenmeyer flask in the laboratory, with the rearing medium maintained at 25 ± 1.0 °C and aeration provided for oxygen supply. During rearing, *C. vulgaris* was provided daily as feed. *D. magna* neonates aged 24 h were harvested for use as test animals, following previous studies [16,43].

Dried brine shrimp eggs (*Artemia salina* L.; SWORM, Seoul, Republic of Korea) were placed in a 250 mL flask containing filtered seawater (32 ppt), with the density of dry brine shrimp eggs set at 1.0 g egg/L. The flasks were incubated at 20 °C with a constant light intensity of 40 μmol/m^2^/s and slight aeration. *Artemia salina* was harvested using a Pasteur pipette 24 h post-hatching for bioassays [16,27].

Lettuce seeds (*Lactuca sativa* L.; Korean cultivar, Hongbitjeokchimasangchu) were purchased from Danong Co., Ltd., Namyangju, Republic of Korea. Seeds were selected based on their intact profile, undamaged condition, and uniform size for growth and survival monitoring.

*Chlorella vulgaris* Beijerinck culture (FBCC-A49) was obtained from the Bioresources Culture Collection, Sangju, Republic of Korea, and divided into two portions for cultivation in freshwater and seawater (32 ppt) medium. A 250 mL Erlenmeyer flask containing 150 mL of Bold’s Basal Medium (BBM) was used as the culture medium [35,42]. Dense cultures were diluted with new sterile BBM every four days to maintain exponential growth. Cultivation took place in a shaking incubator (VS-8480; KOLAB, Korea Science Ltd., Uiwang, Republic of Korea) at 150 rpm, 23.0 ± 1.0 °C, with a light: dark cycle of 14:10 and a light intensity of 4000 lx, under aseptic conditions to avoid contamination.

### 5.2. Catechin Hydrate and Catechin Extracts

Catechin hydrate with 98% purity (HPLC grade) was purchased from Sigma Aldrich, St. Louis, MO, USA. Crude catechins used in this study were extracted from food-grade semi-purified green tea extract powder (Anhui Redstar Pharmaceutical Corp., Ltd., Xuan, Anhui, China) following the method reported by Vuong et al. [44]. Briefly, green tea powder was extracted into a solvent, concentrated, separated, isolated, and dried to produce crude catechins in powder form for further analysis. Catechin hydrate and crude catechin extracts at different concentrations were used to treat *A. salina* nauplii, *D. magna* neonates, *L. sativa* seeds, and *C. vulgaris*. The determination of concentration was based on the preliminary experiments. Crude catechin showed a lower toxic level compared to catechin hydrate. The effects on each organism varied, as did the duration of exposure required. Based on these factors, the concentration applied to arthropods and the observation period differed from those applied to *L. sativa* and *C. vulgaris.*

### 5.3. HPLC Analysis

The catechin content in the green tea extract from Anhui Redstar Pharmaceutical Corporation was analyzed using a 2424 Evaporative Light Scattering (ELC) Detector integrated with an Alliance HPLC System (Waters Corp., Milford, MA, USA) and a Platinum EPS C18 reversed-phase column (53 × 7 mm, 1.5 μm) from Analytical (Flanders, NJ, USA). Standard catechin was sourced from Sigma Aldrich, St. Louis, MO, USA. The mobile phase comprised water and acetonitrile at an 87:13 ratio, with 0.05% (*v*/*v*) trifluoroacetic acid. Samples were filtered through a 0.20 µm syringe filter (Agilent Technologies, Inc., Santa Clara, CA, USA) prior to injection, and the mobile phase was filtered with a 0.45 µm membrane filter (Agilent Technologies) before use. Catechin detection was performed at 210 nm. The sample injection volume was set at 20 µL with a flow rate of 1.0 mL/min, and results were quantified by comparing them with those of the standard solution.

### 5.4. A. salina and D. magna Lethality Test

The *A. salina* and *D. magna* were placed in 24-well plates modified to supply oxygen, based on a previous study by Harwanto et al. [16], as an external oxygen supply is necessary at high densities. The mortality test method for *D. magna* and *A. salina* in this study was based on previous similar studies [16,27,43], with slight modifications. The lethality test was performed to determine the toxic effects of exposure to crude catechins and catechin hydrate on *D. magna* and *A. salina*.

Twenty *D. magna* neonates were placed in each well containing 400 µL of distilled freshwater media, while 20 *A. salina* nauplii were placed in each well containing 400 µL of sterilized seawater media. Concentrations of crude catechins and catechin hydrate were 0.5, 1.0, 2.0, and 3.0 mg/mL for crude catechins and 0.01, 0.05, 0.1, and 0.5 mg/mL for catechin hydrate. Controls without crude catechin or catechin hydrate were also included. The media volume in each well was adjusted to 2 mL with freshwater or seawater [27]. Plates were maintained at 24 ± 1 °C. Survival rate observations were conducted at 24, 48, and 72 h. All experiments were repeated three times.

The survival rate was based on criteria specified by Kim and Choi [27] and Harwanto et al. [16]. *Daphnia magna* neonates and *A. salina* nauplii were considered dead if they showed no movement during observation. Concentration was classified as non-larvicide if the survival rate was >50%, mild larvicidal if >25% but <50%, highly larvicidal if <25%, and extremely larvicidal if no organisms survived.

### 5.5. L. sativa Germination Assays

The germination analysis followed the methods of Choi and Choi [41] and Harwanto et al. [16]. Filter papers with a diameter of 55 mm (Toyo Roshi Kaisha, Ltd., Tokyo, Japan) were placed in 6 cm-diameter sterile Petri dishes and treated with crude catechin or catechin hydrate at concentrations of 1.0, 10.0, and 50.0 mg/mL. The filter papers were then air-dried to ensure that the toxic properties were derived solely from the catechins absorbed on the filter paper.

Ten intact, uniform *L. sativa* seeds with no broken parts were evenly placed on each filter paper and saturated with 1% Tween 20 (L33109; Duksan Pure Chemicals, Ansan, Republic of Korea). Controls were treated with only 1% Tween 20 [45,46]. Since Tween 20 is non-toxic, it was utilized as a surfactant in this study [16]. The plates were placed in an incubator at 25 ± 1 °C in the dark, and germination rates and radicle length were recorded every 24 h for four days. Seeds were considered germinated when the radicle length was 1 mm long [16], measured by a digital caliper. The survival rate of the seeds was determined based on the percentage of germinated seeds.

### 5.6. Yield Inhibition Test of C. vulgaris

The yield inhibition test was based on previous studies by Qian et al. [35] and Xiong et al. [47], with slight modifications. *Chlorella vulgaris* was placed in 15 mL vials containing BBM medium with various concentrations of crude catechin (0.5, 1.0, and 2.0 mg/mL) or catechin hydrate (0.05, 0.1, 0.5, and 1.0 mg/mL). Control vials contained BBM solution without crude catechin or catechin hydrate. Each experiment was repeated three times. Vials were incubated for 72 h under aseptic conditions to avoid contamination. The optical density (OD) was evaluated at 24, 48, and 72 h at 680 nm (OD_680_) using a SPECTROstar^®^ Nano spectrophotometer (BMG LABTECH, Offenburg, Germany).

Cell density (cells/mL) was measured using a hemocytometer (HSU-0650010; Paul Marienfeld GmbH & Co. KG., Lauda-Königshofen, Germany), under a microscope. *Chlorella vulgaris* with a high cell density was diluted to examine the association between optical density and cell density. The linear relationship between OD_680_ and cell density was determined by Equation (1).

Cell density = [(OD_680_ + 0.0282)/0.0004] × 10,000 (*R*^2^ = 0.9936) (1)

The relationship between OD_680_ and dry cell weight (DCW) was also determined following Equation (2) [16,35].

DCW = 0.1244 + 680 + 0.017 (*R*^2^ = 0.9908) (2)

The yield inhibition rate (I_y_; %) was calculated following Equation (3), as reported by Xiong et al. [47].

% I_y_ = ((N_c_ − N_t_)/N_c_) × 100 (3)

where N_c_ is the DCW in the control group and N_t_ is the DCW in the treatment group.

### 5.7. Statistical Analysis

Data are presented as mean ± standard deviation (S.D.) of three independent experiments (*n* = 3). Experimental results were checked for normality and homogeneity before performing the Student’s *t*-test analysis. The *t*-test was used to compare each treatment group with the control group, with statistical significance set at *p* < 0.05. The LC-50, which is the effective toxicant concentration capable of killing 50% of the organisms, was determined using probit analysis. This involved deriving the LC-50 value through regression of the exposed solution concentration against the percentage of organism mortality [48].

## Figures and Tables

**Table 1:** Larvicidal effects of crude catechin and catechin hydrate on the survival rate of *D. magna* neonates.

Incubation Time (h)	Survival Rate (%) of *Daphnia magna* Straus Neonates					LC-50 (µg/mL)
	Control	Crude Catechin (mg/mL)				
		0.5	1.0	2.0	3.0	
24	100.00 ± 0.00	73.00 ± 2.90 *	64.00 ± 6.30 *	45.00 ± 4.10 *	10.00 ± 4.10 *	1174
48	94.00 ± 7.50	43.00 ± 8.70 *	10.00 ± 8.20 *	0.00 ± 0.00 *	0.00 ± 0.00 *	
72	85.00 ± 7.10	28.00 ± 5.00 *	4.00 ± 2.50 *	0.00 ± 0.00 *	0.00 ± 0.00 *	
**Incubation Time (h)**	**Control**	**Catechin Hydrate (mg/mL)**				**LC-50** **(µg/mL)**
		**0.01**	**0.05**	**0.1**	**0.5**	
24	100.00 ± 0.00	88.00 ± 2.90 *	68.00 ± 7.60 *	38.00 ± 2.90 *	0.00 ± 0.00 *	54
48	92.00 ± 2.90	70.00 ± 5.00 *	55.00 ± 5.00 *	28.00 ± 2.90 *	0.00 ± 0.00 *	39
72	77.00 ± 2.90	40.00 ± 5.00 *	20.00 ± 5.00 *	7.00 ± 2.90 *	0.00 ± 0.00 *	7

Values are mean ± S.D. of at least three replications, where each replication contains 20 entities. LC-50: lethal concentration 50, the effective toxicant concentration capable of killing 50% of the organisms (confidence level of 95%). * Indicates significant differences based on Student’s *t*-test at *p* < 0.05 compared to control.

**Table 2:** Larvicidal effects of crude catechin and catechin hydrate on the survival rate of *A. salina* nauplii.

Incubation Time (h)	Survival Rate (%) of *Artemia salina* L. Nauplii					LC-50 (µg/mL)
	Control	Crude Catechin (mg/mL)				
		0.5	1.0	2.0	3.0	
24	100.00 ± 0.00	100.00 ± 0.00	99.00 ± 2.50	54.00 ± 8.50 *	16.00 ± 4.80 *	1895
48	98.00 ± 2.90	85.00 ± 4.10 *	20.00 ± 4.10 *	0.00 ± 0.00 *	0.00 ± 0.00 *	
72	93.00 ± 2.90	33.00 ± 8.70 *	13.00 ± 6.50 *	0.00 ± 0.00 *	0.00 ± 0.00 *	
**Incubation Time (h)**	**Control**	**Catechin hydrate (mg/mL)**				**LC-50** **(µg/mL)**
		**0.01**	**0.05**	**0.1**	**0.5**	
24	100.00 ± 0.00	90.00 ± 0.00 *	63.00 ± 2.90 *	45.00 ± 5.00 *	40.00 ± 5.00 *	153
48	97.00 ± 2.90	80.00 ± 2.90 *	52.00 ± 7.60 *	37.00 ± 5.80 *	28.00 ± 2.90 *	70
72	83.00 ± 2.90	42.00 ± 2.90 *	23.00 ± 2.90 *	10.00 ± 10.00 *	0.00 ± 0.00 *	9

Values are mean ± S.D. of at least three replications, where each replication contains 20 entities. LC-50: lethal concentration 50, the effective toxicant concentration capable of killing 50% of the organisms (confidence level of 95%). * Indicates significant differences based on Student’s *t*-test at *p* < 0.05 compared to control.

**Table 3:** Inhibitory effects of crude catechin and catechin hydrate on the germination rate (%) and radical length (mm) of *L. sativa* seeds.

Incubation Time (h)	Germination Rate (%) of *Lactuca sativa* L. Seeds (Radicle Length of *L. sativa* Seeds in mm)							
	Control		Crude Catechin (mg/mL)					
			1.0		10.0		50.0	
24	82.50 ± 12.60		87.50 ± 15.00		35.00 ± 10.00 *		0.00 ± 0.00 *	
	(3.84 ± 0.46)		(3.11 ± 0.50)		(1.95 ± 0.39 *)		(0.00 ± 0.00 *)	
48	90.00 ± 8.16		95.00 ± 10.00		55.00 ± 5.80 *		0.00 ± 0.00 *	
	(11.34 ± 2.66)		(7.53 ± 1.25)		(3.08 ± 0.41 *)		(0.00 ± 0.00 *)	
72	95.00 ± 5.80		97.50 ± 5.00		75.00 ± 5.80 *		0.00 ± 0.00 *	
	(17.72 ± 3.57)		(12.67 ± 2.27)		(3.64 ± 0.56 *)		(0.00 ± 0.00 *)	
96	95.00 ± 5.80		97.50 ± 5.00		75.00 ± 5.80 *		0.00 ± 0.00 *	
	(23.61 ± 4.64)		(18.02 ± 3.42)		(4.71 ± 1.03 *)		(0.00 ± 0.00 *)	
**Incubation Time (h)**	**Control**	**Catechin Hydrate (mg/mL)**						
		**0.1**		**0.5**	**1.0**	**5.0**		**10.0**
24	83.30 ± 5.80	73.30 ± 5.80		73.30 ± 5.80	70.00 ± 10.00	60.00 ± 17.30		16.70 ± 11.60 *
	(4.10 ± 0.43)	(4.47 ± 0.07)		(4.79 ± 0.55)	(4.42 ± 0.37)	(2.95 ± 0.41 *)		(1.89 ± 0.08 *)
48	90.00 ± 10.00	83.30 ± 5.80		80.00 ± 10.00	70.00 ± 10.00	73.30 ± 20.80		33.30 ± 15.30 *
	(15.74 ± 1.60)	(13.26 ± 0.62 *)		(13.40 ± 1.48)	(12.95 ± 2.31)	(4.33 ± 0.56 *)		(2.40 ± 0.39 *)
72	93.30 ± 5.80	83.30 ± 5.80		86.70 ± 5.80	83.30 ± 15.30	90.00 ± 10.00		33.30 ± 15.30 *
	(24.54 ± 2.48)	(21.93 ± 1.84)		(20.80 ± 3.52)	(15.56 ± 1.31 *)	(5.85 ± 0.92 *)		(2.63 ± 0.60 *)
96	93.30 ± 5.80	83.30 ± 5.80		90.00 ± 0.00	83.30 ± 15.30	90.00 ± 10.00		33.30 ± 15.30 *
	(33.03 ± 1.56)	(29.75 ± 1.00 *)		(26.53 ± 1.23 *)	(20.23 ± 3.14 *)	(6.08 ± 0.27 *)		(2.72 ± 0.62 *)

Values are mean ± S.D. of at least three replications, where each replication contains 10 entities. * Indicates significant differences based on Student’s *t*-test at *p* < 0.05 compared to control.

**Table 4:** Toxicity of crude catechin and catechin hydrate on the cell density of freshwater *C. vulgaris*.

Incubation Time (h)	Cell Density of Freshwater *Chlorella vulgaris* Beijerinck (10^6^ cells/mL)						
	Control	Crude Catechin (mg/mL)					
		0.5		1.0		2.0	
0	1.08 ± 0.02	1.05 ± 0.06		1.05 ± 0.06		1.06 ± 0.01	
24	1.16 ± 0.05	1.50 ± 0.04 *		1.42 ± 0.06 *		1.17 ± 0.06	
48	2.02 ± 0.08	2.33 ± 0.07 *		2.27 ± 0.07 *		1.50 ± 0.04 *	
72	3.71 ± 0.24	3.84 ± 0.10		3.31 ± 0.22 *		1.58 ± 0.05 *	
96	4.26 ± 0.14	4.60 ± 0.17 *		3.40 ± 0.08 *		1.90 ± 0.06 *	
Yield inhibition		−(4.61) ± 0.83%		12.29 ± 1.60%		33.84 ± 0.60%	
**Incubation Time (h)**	**Control**	**Catechin Hydrate (mg/mL)**					
		**0.05**	**0.1**		**0.5**		**1.0**
0	1.11 ± 0.05	1.13 ± 0.04	1.10 ± 0.06		1.12 ± 0.06		1.15 ± 0.05
24	1.28 ± 0.04	1.59 ± 0.10 *	1.56 ± 0.29 *		1.56 ± 0.07 *		1.33 ± 0.02 *
48	2.02 ± 0.09	2.22 ± 0.27	2.09 ± 0.28		2.39 ± 0.04 *		1.64 ± 0.06 *
72	3.56 ± 0.22	3.99 ± 0.33 *	3.61 ± 0.35		2.82 ± 0.07 *		1.64 ± 0.05 *
96	4.04 ± 0.18	4.74 ± 0.16 *	4.28 ± 0.18 *		3.10 ± 0.06 *		1.68 ± 0.05 *
Yield inhibition		−(8.10) ± 1.22%	−(2.91) ± 1.03%		13.85 ± 1.53%		34.91 ± 1.26%

Values are mean ± S.D. of at least three replications. * Indicates significant differences based on Student’s *t*-test at *p* < 0.05 compared to control.

**Table 5:** Toxicity of crude catechin and catechin hydrate on the cell density of seawater *C. vulgaris*.

Incubation Time (h)	Cell Density of Seawater *Chlorella vulgaris* Beijerinck (10^6^ cells/mL)				
	Control	Crude Catechin (mg/mL)			
		0.5	1.0		2.0
0	1.06 ± 0.04	1.02 ± 0.04	1.04 ± 0.04		1.06 ± 0.05
24	1.18 ± 0.04	1.22 ± 0.04	1.36 ± 0.18		1.23 ± 0.08
48	1.77 ± 0.08	1.90 ± 0.08 *	1.97 ± 0.13 *		1.37 ± 0.25 *
72	3.06 ± 0.21	3.52 ± 0.26 *	2.49 ± 0.25 *		1.55 ± 0.20 *
96	3.96 ± 0.19	4.25 ± 0.19 *	2.63 ± 0.19 *		1.61 ± 0.16 *
Yield inhibition		−(2.61) ± 1.21%	19.90 ± 2.29%		35.26 ± 1.06%
**Incubation Time** **(h)**	**Control**	**Catechin Hydrate (mg/mL)**			
		**0.05**	**0.1**	**0.5**	**1.0**
0	1.12 ± 0.07	1.15 ± 0.06	1.09 ± 0.04	1.15 ± 0.09	1.12 ± 0.13
24	1.25 ± 0.04	1.36 ± 0.11 *	1.56 ± 0.22 *	1.35 ± 0.09 *	1.32 ± 0.08 *
48	2.29 ± 0.08	2.21 ± 0.10	2.20 ± 0.17	1.91 ± 0.12 *	1.46 ± 0.09 *
72	3.35 ± 0.16	3.57 ± 0.14 *	3.41 ± 0.25	2.28 ± 0.13 *	1.72 ± 0.08
96	3.87 ± 0.29	4.15 ± 0.16 *	3.98 ± 0.18	2.77 ± 0.09 *	1.50 ± 0.09 *
Yield inhibition		−(5.92) ± 2.18%	−(1.71) ± 2.21%	16.37 ± 1.53%	35.97 ± 1.70%

Values are mean ± S.D. of at least three replications. * Indicates significant differences based on Student’s *t*-test at *p* < 0.05 compared to control.

## Data Availability

The original contributions presented in this study are included in this article. Further inquiries can be directed to the corresponding authors.

## Abbreviations

The following abbreviations are used in this manuscript:

LC-50	Lethal concentration 50, the effective toxicant concentration capable of killing 50% of the organisms
DCW	Dry cell weight
OD	Optical density
BBM	Bold’s Basal Medium
HPLC	High-performance liquid chromatography
EC	Epicatechin
EGC	Epigallocatechin
ECG	Epicatechin gallate
EGCG	Epigallocatechin gallate
